# Organized community sport participation for children and youth with physical disability: A scoping review protocol

**DOI:** 10.1371/journal.pone.0332784

**Published:** 2026-06-15

**Authors:** Karen Davies, Mitchell Barran, Colleen Pawliuk, Tiago Choi, Bruna Schilbach Pizzutto, Karen Lai, Patricia Moreno Grangeiro, Courtney L. Pollock

**Affiliations:** 1 The Motion Lab, Sunny Hill Health Centre at BC Children’s Hospital, Vancouver, British Columbia, Canada; 2 Faculty of Medicine, Department of Physical Therapy, University of British Columbia, Vancouver, British Columbia, Canada; 3 Faculty of Medicine, University of British Columbia, Vancouver, British Columbia, Canada; 4 Faculty of Medicine, Department of Pediatrics, University of British Columbia, Vancouver, British Columbia, Canada; 5 Faculdade de Medicina, Universidade de São Paulo, São Paulo, São Paulo, Brazil; 6 Community Partner, Vancouver, British Columbia, Canada; 7 Instituto de Ortopedia e Traumatologia, Hospital das Clínicas HCFMUSP, São Paulo, São Paulo, Brazil; 8 Rehabilitation Research Program, Centre for Aging SMART, Vancouver Coastal Health Research Institute, Vancouver, British Columbia, Canada; Arba Minch University, ETHIOPIA

## Abstract

Children and youth living with physical disability have less opportunities to participate in organized meaningful physical activities in their communities than their typically developing peers. Equitable access to community sport programs that aim to support the health, social participation, and well-being of these children and youth is required. Frameworks for program implementation and evaluation offer practical tools for supporting quality participation. Limited research exists demonstrating the practical application of these conceptual model frameworks. The objective of this scoping review is to identify evidence of evaluation of organized community sport participation opportunities for children and youth with physical disabilities through the lens of the RE-AIM and Quality Parasport Participation Frameworks. This review will consider studies that explore organized community-based sport participation for children and youth up to 25 years with physical disability without limitation to specific geographic area. Children and youth with only a cognitive or intellectual disability, or the absence of a physical disability, will be excluded. The databases to be searched include MEDLINE (Ovid), Embase (Ovid), SportDiscus (EBSCO), CINAHL (EBSCO), PEDro, Global Index Medicus and Google Scholar. Sources of grey literature will also be searched. Anticipated limitations are the exclusion of clinical intervention trials and the exclusion of unorganized sport. The latter may limit cultural relevance and global scope, as informal sports could dominate in this context and be underrepresented in the literature. Languages will also be restricted to English, French, Portuguese and Spanish. No publication date restrictions will be placed on the search. Two independent reviewers will perform title and abstract screening, and retrieval and assessment of full-text citations. Any disagreements that arise between the reviewers at each stage of the selection process will be resolved through discussion or with a third reviewer. A narrative synthesis of the results will be presented in conjunction with a tabular and/or graphic format.

## Introduction

Every child has the right to participate in sports, yet it can be challenging for children and youth with physical disabilities to find meaningful sport participation opportunities in their community. The European Sports Charter defines sport as “all forms of physical activity which, through casual or organized participation, are aimed at maintaining or improving physical fitness and mental well-being, forming social relationships or obtaining results in competition at all levels” [1]. Within this definition, our review will focus on organized sports involving structured programs with regular planned practice and defined objectives (e.g., typically including skill development, physical fitness, teamwork, recreation and/or competition). Participation in sport provides health benefits and improves health outcomes for children and youth with physical disabilities [2], promoting achievement of fine and gross motor skills, sport-specific physical ability, increased fitness, mobility, muscular strength, and opportunities for social network development [3–5]. Positive social experiences from participation in sport for youth with physical disability is defined more comprehensively by the Quality Parasport Participation Framework (QPPF) [6]. This framework integrates insights from several theoretical underpinnings to define optimal or quality participation incorporating unique social benefits and well documented facilitators and barriers that impact physical activity and sport participation in this population [6–8]. Despite the work done by this group, limited evaluation of current community sport programs indicates the need for program implementation and evaluation frameworks to support quality participation for children and youth with physical disability.

The World Health Organization (WHO) describes disability as an inherent part of the human experience; the outcome of the interaction between an experienced health condition and/or impairment and circumstances associated with the environment and personal factors [9]. Within the context of the International Classification of Functioning, Disability and Health (ICF), a physical disability is a health condition or physical impairment that may limit activity or restrict participation [10]. The minimum estimated global prevalence of children and adolescents younger than 20 years living with a disability is between 266 to 291.3 million (10.1–11.3%) [11]. For youth ages 15–24 years, the prevalence is less clear. Children and youth with disabilities experience significant gaps in sport participation, taking part in less physical activity, recreation, and sport than their typically developing peers, and are less likely to meet international guidelines for physical activity [5,12–16]. Further, children and adolescents with disability participate less (and with less diversity, frequency, and intensity) in team or non-team sports when compared to their peers with typical development [15,17]. They are also more likely to play informal or casual games at home than more formal or organized sports in their community, even if sports are their preferred activities [15,17].

The Canadian Disability Participation Project (CDPP) is a national organization that aims to improve quality participation, differentiating from merely recording attendance, in community-based sport, optimizing outcomes of participation among children, youth and adults experiencing disability [18]. They developed the QPPF to identify and describe quality experience in sport inclusive of six building blocks: autonomy, belongingness, challenge, engagement, mastery, and meaning [6,19]. These building blocks are aligned with the barriers and facilitators of community-based sport participation for children and youth with physical disabilities and can be evaluated at the physical, program, and social environment levels. The RE-AIM Framework - Reach for intended population; Effectiveness of implementation; Adoption by community; Implementation consistency, costs, adaptations; and Maintenance of intervention effects – has also been utilized to provide a comprehensive evaluation of public health programs [20]. Informed by this framework, Lawrason et al [21] have created a toolkit for applying the dimensions of RE-AIM in local community-based physical activity contexts for evidence-informed program evaluation.

A recent systematic review with meta-analysis identifying health outcomes for children and youth with physical disability participating in sports and physical recreation has identified several gaps in the literature [2]. They found that existing quantitative studies focused more on therapeutic and impairment models. Investigation of outcomes focused on participation occurring in community settings and more natural environments, to align with the goal of increasing health and inclusion for this population, is warranted [2]. Primarily most population data describing physical activity participation in children and youth with disability also derives from high-income countries [22]. Evidence on the influence of physical activity among children and youth with physical disability remains limited worldwide [2,22].

To our knowledge, no reviews have included an additional grey literature search on this topic to capture programs in middle- or lower-income countries and identify programs that already exist in communities around the world. A preliminary search of PROSPERO, MEDLINE, Open Science Framework and Google Scholar was conducted, and no current or in-progress scoping reviews or systematic reviews on the topic were identified. Therefore, to further address the current gaps in the literature, the purpose of this scoping review is to systematically map and synthesize the level of evidence related to program evaluation of community sport participation in children and youth living with physical disability. A secondary aim is to further map the perceived barriers and facilitators for community sport program implementation to the QPPF and RE-AIM framework.

### Review questions

1. What is the level of evidence related to program evaluation of community sport participation opportunities for children and youth up to 25 years of age with physical disabilities?
2. What are the perceived barriers and facilitators for community sport program implementation when mapped to the QPPF and RE-AIM framework?

## Materials and methods

### Inclusion and exclusion criteria

#### Participants.

This scoping review will consider studies that include children and youth up to 25 years of age with physical disabilities. This age cutoff is consistent with the WHO and United Nations’ definitions of youth [23,24]. Studies with participants (adults) outside of this age range will be included if at least 50% of the population sample is less than 25 years of age. Children and youth with only a cognitive or intellectual disability, or the absence of a physical disability, will be excluded. Studies including mixed-disability cohorts, where physical, sensory and/or cognitive disabilities coexist, will be included if at least 50% of the population sample have a primary physical disability or sensory impairment. For this review, physical disability is defined broadly as the outcome of the interaction between a child or youth’s long-term physical health condition and their personal and environmental factors [9]. When evaluating community sport programs, we will include children and youth, family and caregivers, coaches, program administrators, volunteers, healthcare and recreational professionals as well as any person providing support to the participants or the program.

#### Concept.

This review will consider studies that explore the evaluation of community sport participation for children and youth with physical disabilities. If explicit reporting of program evaluation is absent, we will look for any dimensions that fall within the context of the RE-AIM framework and/or QPPF. Studies that include para-athletics, adaptive sports, and integrated programs in mainstream sports will be evaluated correspondingly through the lens of the QPPF and RE-AIM framework. Studies that describe adapted equipment, injury management, or para-athletics classification without addressing participation will be excluded. Unorganized physical activity, leisure and recreation studies will also be excluded. Barriers and facilitators to participation will be mapped to the RE-AIM framework and the QPPF. They will be assessed quantitatively in terms of frequency in relation to the RE-AIM framework and qualitatively in accordance with the themes described in the QPPF.

#### Context.

This review will consider studies that implement organized sport participation programs for children and youth with physical disability in the community. Community settings will not be limited to specific geographical areas. They may include but will not be limited to indoor or outdoor recreation centres, fields, sports clubs, gymnasiums, and aquatic centres. We will exclude studies that take place at home, in a clinical setting or school-based therapy interventions. Organized sports participation in a school setting will also be excluded due to underlying differences in program structure. Hybrid therapy (clinical) and sport programs will be included if there are community sport-related goals or if the aim is to support community sport participation.

### Types of sources

This scoping review will consider primary data in the form of quantitative, qualitative, and mixed methods study designs. Systematic or narrative reviews, un-published text, opinion papers, editorials and protocols and/or clinical trial registries that do not include results will be excluded.

### Methodology

The proposed scoping review will be conducted in accordance with the Joanna Briggs Institutes (JBI) methodology for scoping reviews [25] and will be reported in line with the Preferred Reporting Items for Systematic Reviews and Meta-Analyses extension for Scoping Reviews (PRISMA-ScR) [26]. This protocol was developed in accordance with PRISMA for Protocols [27] (S1 Checklist).

### Search strategy

The search strategy will aim to locate both published and unpublished primary studies. An initial limited search of MEDLINE (Ovid) and SportDiscus (EBSCO) was undertaken to identify articles on the topic (S2 Fig). The text words contained in the titles and abstracts of relevant articles, and the index terms used to describe the articles, were used to develop a full search strategy for MEDLINE (Ovid; S3 Table). The search strategy, including all identified keywords and index terms, will be adapted for each included information source. The key words and index terms included were based on the experience of the authors and preliminary testing of the index terms. The reference lists of articles included in the review will be screened for additional papers using Web of Science Core Collection.

Articles published from database inception to present will be included. We will search MEDLINE (Ovid), Embase (Ovid), SportDiscus (EBSCO), CINAHL (EBSCO), PEDro, Global Index Medicus and Google Scholar. Sources of grey literature to be searched include ProQuest Dissertations & Theses Citation Index (Web of Science), Open Access Theses and Dissertations (OATD), Papers First (WorldCat FirstSearch), Proceedings (WorldCat FirstSearch), PolicyCommons, Google, Biblioteca Virtual de Saúde (BVS) and Biblioteca Digital de Teses e Dissertações (BDTD).

### Study/Source of evidence selection

Following the search, all identified records, including grey literature, will be collated and uploaded into Covidence (Veritas Health Innovation, Melbourne, Australia) and duplicates removed. Following a pilot test, titles and abstracts will then be screened by two independent reviewers for assessment against the inclusion criteria for the review. Potentially relevant papers will be retrieved in full. The full text of selected citations will be assessed in detail against the inclusion criteria by two independent reviewers. Reasons for exclusion of full-text papers that do not meet the inclusion criteria will be recorded and reported in the scoping review. Any disagreements that arise between the reviewers at each stage of the selection process will be resolved through discussion or with a third reviewer. The results of the search will be reported in full in the final scoping review and presented in a PRISMA flow diagram [28]. We will include studies in English, French, Portuguese and Spanish in line with author fluency. This will be addressed as a limitation in the final manuscript.

### Data extraction

Data will be extracted from papers included in the scoping review by two independent reviewers using a data extraction tool developed by the reviewers. The data extracted will include specific details about the study design and methods, participants, sport and context characteristics [29], outcomes defined by the RE-AIM framework and QPPF and associated barriers and facilitators of implementation, integration models, and other key elements relevant to the review questions. Themes or barriers specific to sex and/or gender will be captured in the data extraction tool and described. A draft extraction tool is provided (S4 Table). The draft data extraction tool will be modified and revised as necessary during the process of extracting data from each included paper. Modifications will be detailed in the full scoping review. Any disagreements that arise between the reviewers will be resolved through discussion or with a third reviewer. Authors of papers will be contacted to request missing or additional data, where required.

### Critical appraisal

All eligible studies included in the review will be critically appraised by two independent reviewers for methodological quality in the review using standardized critical appraisal instruments from JBI for experimental, quasi-experimental, observational and qualitative studies. A pilot test will be conducted with five studies to ensure consistency. If a team member has authored any studies included in our review, they will abstain from critical appraisal. Any disagreements that arise between the reviewers will be resolved through discussion or with a third reviewer. The results of critical appraisal will be reported in a table with an accompanying narrative.

## Results and discussion

### Data analysis and presentation

We will share a narrative synthesis of the results and tables and/or graphs as appropriate in accordance with the review objectives and questions. All data underlying the scoping review (including search strategies, reference files at each stage and full data extraction and critical appraisal) will be deposited in the Open Science Framework: https://osf.io/dsny4.

## Supporting information

S1 Checklist PRISMA-P 2015 Checklist. (DOCX)

S2 Fig Search Structure. (DOCX)

S3 Table Search Strategy. (DOCX)

S4 Table Data Extraction Instrument. (DOCX)
