# Supplementary material for: Organized community sport participation for children and youth with physical disability: A scoping review protocol
Source: PLoS One. 2026 Jun 15;21(6):e0332784. doi: 10.1371/journal.pone.0332784 (PMC13268187; doi:10.1371/journal.pone.0332784)
Supplement: S2 Fig — (DOCX) [file pone.0332784.s002.docx]

**Supporting Information**

# S2 Fig. Search Structure.
