# Supplementary material for: Organized community sport participation for children and youth with physical disability: A scoping review protocol
Source: PLoS One. 2026 Jun 15;21(6):e0332784. doi: 10.1371/journal.pone.0332784 (PMC13268187; doi:10.1371/journal.pone.0332784)
Supplement: S3 Table — (DOCX) [file pone.0332784.s003.docx]

**Supporting Information**

# S3 Table. Search Strategy.

# Ovid MEDLINE(R) ALL <1946 to June 06, 2025>

| **#** | **Query** | **Results from 9 Jun 2025** |
| --- | --- | --- |
| 1 | Children with Disabilities/ | 7,447 |
| 2 | (disabil* or impairment?).tw,kf. | 733,740 |
| 3 | Cerebral Palsy/ | 25,262 |
| 4 | (cerebral palsy or spastic diplegia).tw,kf. | 30,759 |
| 5 | Meningomyelocele/ | 4,726 |
| 6 | (meningomyelocele or spina bifida).tw,kf. | 10,023 |
| 7 | (neurological adj2 (diseas* or disab* or disorder?)).tw,kf. | 80,202 |
| 8 | Brain Injuries, Traumatic/ | 16,359 |
| 9 | acquired brain injur*.tw,kf. | 3,595 |
| 10 | exp Limb Deformities, Congenital/ | 27,236 |
| 11 | (limb deformit* or limb deficienc*).tw,kf. | 2,012 |
| 12 | exp Joint Diseases/ | 457,092 |
| 13 | Legg-Calve-Perthes Disease/ | 2,053 |
| 14 | "Developmental Dysplasia of the Hip"/ | 638 |
| 15 | (joint disease? or joint deficienc* or Legg-Calve-Perthes Disease or "Developmental Dysplasia of the Hip" or congenital hip dysplasia or developmental hip dysplasia).tw,kf. | 20,445 |
| 16 | Spinal Cord Injuries/ | 47,397 |
| 17 | (spinal cord Injur* or post-traumatic myelopathy).tw,kf. | 53,207 |
| 18 | or/1-17 | 1,383,197 |
| 19 | (sports/ or baseball/ or basketball/ or bicycling/ or boxing/ or cricket sport/ or football/ or gaelic football/ or golf/ or gymnastics/ or hockey/ or martial arts/ or tai ji/ or mountaineering/ or racquet sports/ or tennis/ or return to sport/ or rugby/ or running/ or jogging/ or marathon running/ or skating/ or snow sports/ or skiing/ or soccer/ or sports for persons with disabilities/ or team sports/ or lacrosse/ or "track and field"/ or volleyball/ or walking/ or nordic walking/ or water sports/ or swimming/ or diving/ or weight lifting/ or wrestling/ or youth sports/) and (community or adapt* or organized or program* or modif* or para or intervention?).tw,kf. | 47,771 |
| 20 | ((sport? or sporting or athletics or baseball or basketball or bicycling or cycling or boxing or cricket or football or golf or gymnastics or hockey or martial arts or tai ji or tai chi or hiking or tennis or rubgy or running or jogging or skating or skiing or snowboarding or lacrosse or "track and field" or volleyball or walking or swimming or diving or rowing or weight lifting or strength training or wrestling or mobility or stretching) adj5 (community or adapt* or organized or program* or modif* or para or intervention?)).tw,kf. | 29,955 |
| 21 | 19 or 20 | 66,589 |
| 22 | participat*.tw,kf. | 770,851 |
| 23 | exp child/ or exp infant/ or adolescent/ or exp pediatrics/ or child, abandoned/ or exp child, exceptional/ or child, orphaned/ or child, unwanted/ or minors/ or young adult/ or (pediatric* or paediatric* or (child* not childbearing) or newborn* or congenital* or infan* or baby or babies or neonat* or pre-term or preterm* or premature birth* or NICU or preschool* or pre-school* or kindergarten* or kindergarden* or elementary school* or nursery school* or (day care* not adult*) or schoolchild* or toddler* or boy or boys or girl* or middle school* or pubescen* or juvenile* or teen* or youth* or young adult? or young people or high school* or adolesc* or pre-pubesc* or prepubesc*).mp. or (child* or adolesc* or pediat* or paediat*).jn. | 5,994,145 |
| 24 | 18 and 21 and 22 and 23 | 916 |
