# Supplementary material for: Organized community sport participation for children and youth with physical disability: A scoping review protocol
Source: PLoS One. 2026 Jun 15;21(6):e0332784. doi: 10.1371/journal.pone.0332784 (PMC13268187; doi:10.1371/journal.pone.0332784)
Supplement: S4 Table — (DOCX) [file pone.0332784.s004.docx]

**Supporting Information**

**S4 Table. Data Extraction Instrument.**

| Authors, Year of Publication, Country of Origin | Type of Study: Study title, study design | Objective/Research Question | Measured outcome + measurement tools | Population, sex and/or gender, # of Program Participants | Program Description: type of activity, competition level, team size + location | Implementation framework or integration model + associated facilitators/barriers mapped to RE-AIM framework | Participant experience through lens of QPPF | Summary of findings |
| --- | --- | --- | --- | --- | --- | --- | --- | --- |
