# Supplementary material for: Organized community sport participation for children and youth with physical disability: A scoping review protocol
Source: PLoS One. 2026 Jun 15;21(6):e0332784. doi: 10.1371/journal.pone.0332784 (PMC13268187; doi:10.1371/journal.pone.0332784)
Supplement: Attachment — Submitted filename: Response to Reviewers.docx [file pone.0332784.s006.docx]

February 16, 2025

Dear Academic Editor and Reviewer,

We thank the reviewer for their thoughtful and constructive edits and points to consider as well as the opportunity to submit a revised version of our manuscript. Please see our responses below each point raised in the review of the study protocol submission PONE-D-25-48289 “Organized community sport participation in children and youth with physical disability: a scoping review protocol.” Line numbers align with the file labeled ‘Revised Manuscript with Tracked Changes’.

Reviewer #1:

The protocol addresses an important and timely topic with a clear structure and adherence to recognized guidelines (JBI, PRISMA-ScR), which is commendable. However, several aspects require refinement to enhance clarity and rigor. Key terms such as “organized community sport” and “physical disability” need precise operational definitions to avoid ambiguity, and the rationale for the age cutoff (up to 25 years) should be justified. The objectives should explicitly integrate the theoretical frameworks (RE-AIM and QPPF) mentioned later in the text, and the global scope requires more detail on how language diversity, cultural differences, and low-resource settings will be handled. The methods section should provide clearer strategies for grey literature screening, translation, and inter-rater reliability checks. Additionally, the exclusion of unorganized sport may limit cultural relevance, and *anticipated limitations such as heterogeneity and publication bias should be acknowledged.* Minor issues include typographic inconsistencies, citation formatting, and duplicate references. Overall, the protocol is promising but would benefit from stronger conceptual clarity, methodological transparency, and attention to global applicability.

**Response:** We do not believe this scoping review is limited by publication bias. We have conducted a rigorous grey literature search, and we will include all publication types that meet our eligibility criteria.

Specific Comments

Title
How do you operationalize “organized” and “community” in global contexts where informal sports dominate?

**Response:** In the Introduction we have further clarified, The European Sports Charter defines sport as “all forms of physical activity which, through casual or organized participation, are aimed at maintaining or improving physical fitness and mental well-being, forming social relationships or obtaining results in competition at all levels”[1]. **Within this definition, our review will focus on organized sports involving structured programs with regular planned practice and defined objectives (e.g., typically including skill development, physical fitness, teamwork, recreation and/or competition). See lines 35-37.**

To operationalize “community”, the following has been added to the context section in the inclusion and exclusion criteria in Methods**, Lines 148-152: This review will consider studies that implement organized sport participation programs for children and youth with physical disability in the community. Community settings will not be limited to specific geographical areas. They may include but will not be limited to indoor or outdoor recreation centres, fields, sports clubs, gymnasiums, and aquatic centres.** We have removed the comment in Line 150 that there will be a global scope to remove misinterpretation that we will be able to capture a true global scope of community sports participation. Our aim is to focus on formal sports participation for children and youth with physical disability.

Will para-athletics and adaptive sports be treated differently from mainstream sports?

**Response:** Added to the concept section in the inclusion and exclusion criteria in Methods, **Lines 138-140: Studies that include para-athletics, adaptive sports, and integrated programs in mainstream sports will be evaluated correspondingly through the lens of the QPPF and RE-AIM Frameworks.**

Abstract
The abstract lacks explicit mention of frameworks (RE-AIM, QPPF) in objectives, these appear later but should be upfront.

**Response:** Added to the abstract, **Lines 8-11: The objective of this scoping review is to identify evidence of evaluation of organized community sport participation opportunities for children and youth with physical disabilities through the lens of the RE-AIM and Quality Parasport Participation Frameworks.**

Also, no mention of anticipated limitations (e.g., heterogeneity, lack of intervention trials).

**Response:** Added to the abstract, **Lines 19-23:** **Anticipated limitations are the exclusion of clinical intervention trials** **and the exclusion of unorganized sport. The latter may limit cultural relevance and global scope, as informal sports could dominate in this context and be underrepresented in the literature. Languages will also be restricted to English, French, Portuguese and Spanish. No publication date restrictions will be placed on the search.** We expect heterogeneity and consider this a strength.

Why was age cutoff set at 25 years? Is this consistent with WHO youth definition or arbitrary?

**Response:** Added to the participants section in the inclusion and exclusion criteria in Methods, **Lines 120-122:** **This age cutoff is consistent with the WHO and United Nations’ definitions of youth[23,24]. Studies with participants (adults) outside of this age range will be included if at least 50% of the population sample is less than 25 years of age.**

How will you handle mixed-disability cohorts where physical and cognitive disabilities coexist?

**Response:** Added to the participants section in the inclusion and exclusion criteria in Methods, **Lines 124-126:** **Studies including mixed-disability cohorts, where physical, sensory and/or cognitive disabilities coexist, will be included if at least 50% of the population sample have a primary physical disability or sensory impairment.**

Introduction
Heavy reliance on prevalence data without linking to sport participation gaps quantitatively.

**Response:** Added link to this gap in the Introduction, **Lines 61-69: Children and youth with disabilities experience significant gaps in sport participation, taking part in less physical activity, recreation and sport than their typically developing peers and less likely to meet international guidelines for physical activity[5, 12-16]. Further, children and adolescents with disability participate less, and with less diversity, frequency and intensity, in team or non-team sports when compared to their peers with typical development[15,17]. They are also more likely to play informal or casual games at home than more formal or organized sports in their community, even if sports are their preferred activities[15,17].**

Mentions barriers but does not classify them systematically (e.g., socio-economic vs infrastructural).

**Response:** Removed specific barriers from the Introduction, **Lines 48-50**, as these will be included in the data extraction tool and classified systematically in the final manuscript.

Line 62, “Positive social experiences enhance participation”, needs stronger theoretical underpinning (Self-Determination Theory?).

**Response:** Added to the Introduction, **Lines 41-47: Positive social experiences from participation in sport for youth with physical disability is defined more comprehensively by the Quality Parasport Participation Framework (QPPF)[6]. This framework integrates insights from several theoretical underpinnings to define optimal or quality participation incorporating unique social benefits and well documented facilitators and barriers that impact physical activity and sport participation in this population.**

Line 79, How will you differentiate between “quality participation” and mere attendance?

**Response:** Many studies may report attendance and not address a metric that will inform quality participation, and this will be noted. Quality participation will be described and evaluated through the constructs of the QPPF. Added to the Introduction, **Lines 72-78: The Canadian Disability Participation Project (CDPP) is a national organization that aims to improve quality participation, differentiating from merely recording attendance, in community-based sport, optimizing outcomes of participation among children, youth and adults experiencing disability[18]. They developed the Quality Parasport Participation Framework (QPPF) to identify and describe quality experience in sport inclusive of six building blocks: autonomy, belongingness, challenge, engagement, mastery, and meaning[6,19].**

Will cultural factors (e.g., gender norms in sport) be analyzed?

**Response:** We will report participation levels by sex and/or gender in our data extraction tool. Added sex and/or gender as a descriptor under population heading in the data extraction instrument in **S3 Table**.

Review questions
Second question (“barriers and facilitators”) is broad, should specify if these will be mapped to frameworks or thematic synthesis.

**Response:** Added to the second review question in the Introduction**, Lines 113-114: What are the perceived barriers and facilitators for community sport program implementation when mapped to the QPPF and RE-AIM framework?**

Will you assess barriers/facilitators quantitatively (frequency) or qualitatively (themes)?

**Response:** Added to the concept section in the inclusion and exclusion criteria in Methods, **Lines 144-146: Barriers and facilitators to participation will be mapped to the RE-AIM framework and the QPPF. They will be assessed quantitatively in terms of frequency in relation to the RE-AIM framework and qualitatively in accordance with the themes described in the QPPF.**

How will you address intersectionality (e.g., gender + disability)?

**Response:** Added to data extraction in Methods, **Lines 206-207: Themes or barriers specific to sex and/or gender will be captured in the data extraction tool and described.**

Eligibility criteria
“Physical disability” definition is vague, should align with ICD or ICF classification.

**Response:** Added to the Introduction, **Lines 54-59: The World Health Organization (WHO) describes disability as an inherent part of the human experience; the outcome of the interaction between an experienced health condition and/or impairment and circumstances associated with the environment and personal factors[9]. Within the context of the International Classification of Functioning, Disability and Health (ICF), a physical disability is a health condition or physical impairment that may limit activity or restrict participation[10].**

Exclusion of “unorganized sport” may omit culturally relevant practices in LMICs.

**Response:** Added to the context section in the inclusion and exclusion criteria in Methods, **Lines 148-152:** **This review will consider studies that implement organized sport participation programs for children and youth with physical disability in the community. Community settings will not be limited to specific geographical areas. They may include but will not be limited to indoor or outdoor recreation centres, fields, sports clubs, gymnasiums, and aquatic centres.**

Why exclude school-based therapy interventions but include school sports?

**Response:** We will exclude both school-based therapy interventions and school sports. This is added to the context section in the inclusion and exclusion criteria in Methods, **Lines 152-154: We will exclude studies that take place at home, in a clinical setting or school-based therapy interventions. Organized sports participation in a school setting will also be excluded due to underlying differences in program structure.**

How will you handle hybrid programs (therapy + sport)?

**Response:** Added to the context section in the inclusion and exclusion criteria in Methods, **Lines 154-156: Hybrid therapy (clinical) and sport programs will be included if there are community sport-related goals or if the aim is to support community sport participation.**

Methods
Grey literature search strategy is ambitious but lacks detail on screening rigor.

**Response:** Added to the study/source of evidence section in Methods, **Lines 188-197: Following the search, all identified records, including grey literature, will be collated and uploaded into Covidence (Veritas Health Innovation, Melbourne, Australia) and duplicates removed. Following a pilot test, titles and abstracts will then be screened by two independent reviewers for assessment against the inclusion criteria for the review. Potentially relevant papers will be retrieved in full. The full text of selected citations will be assessed in detail against the inclusion criteria by two independent reviewers. Reasons for exclusion of full-text papers that do not meet the inclusion criteria will be recorded and reported in the scoping review. Any disagreements that arise between the reviewers at each stage of the selection process will be resolved through discussion or with a third reviewer. The results of the search will be reported in full in the final scoping review and presented in a PRISMA flow diagram (S1)[28].**

No mention of language translation process, critical for global scope.

**Response:** Added to the study/source of evidence section in Methods, **Lines 198-199: We will include studies in English, French, Portuguese and Spanish in line with author fluency. This will be addressed as a limitation in the final manuscript.**

Will you use dual independent screening for grey literature?

**Response:** Added to the study/source of evidence section in Methods, **Lines 188-191: Following the search, all identified records, including grey literature, will be collated and uploaded into Covidence (Veritas Health Innovation, Melbourne, Australia) and duplicates removed. Following a pilot test, titles and abstracts will then be screened by two independent reviewers for assessment against the inclusion criteria for the review.**

How will you assess methodological quality (JBI critical appraisal tools?) even though scoping reviews typically do not?

**Response:** Added in the Methods, **Lines 214-221, All eligible studies included in the review will be critically appraised by two independent reviewers for methodological quality in the review using standardized critical appraisal instruments from JBI for experimental, quasi-experimental, observational and qualitative studies. A pilot test will be conducted of five studies to ensure consistency. If a team member has authored any studies included in our review, they will abstain from critical appraisal. Any disagreements that arise between the reviewers will be resolved through discussion or with a third reviewer. The results of critical appraisal will be reported in a table with accompanying narrative.**

References
Inconsistent referencing (authors used ‘ …et al’ after mentioning three authors in reference [2], whereas after mentioning six authors in references, [3, 10, 19, 21, …]. Also, eight authors were mentioned in reference [16], reference number [13] and [15] are the same, please check all references in the entire protocol and format according to the journal’s guideline.

**Response:** Referencing has been edited for consistency and in the format according to PLOS ONE’s guidelines.

Supplementary files
Search strategy lacks Boolean logic clarity for complex queries.

**Response:** Our search strategy was designed by a health sciences librarian and is reported in accordance with the PRISMA 2020 guidelines that require a search strategy be reported exactly as run in the database. We have added a diagram to help clarify the complex Boolean logic at play in the strategy for the layperson in S2 Fig Search strategy**.**

Have the authors described where all data underlying the findings will be made available when the study is complete?

Reviewer #1: No

**Response:** Added in Results and Discussion, **Lines 228-232: We will share a narrative synthesis of the results and tables and/or graphs as appropriate in accordance with the review objectives and questions. All data underlying the scoping review (including search strategies, reference files at each stage and full data extraction and critical appraisal) will be deposited in the Open Science Framework: https://osf.io/dsny4.**

Thank-you for considering our scoping review protocol revisions. We look forward to hearing from you.

Sincerely,

Karen Davies

krdavies@cw.bc.ca
