# Supplementary material for: Organized community sport participation for children and youth with physical disability: A scoping review protocol
Source: PLoS One. 2026 Jun 15;21(6):e0332784. doi: 10.1371/journal.pone.0332784 (PMC13268187; doi:10.1371/journal.pone.0332784)
Supplement: Peer review history

# Peer review: Decision Letter 0

**DOI:** 10.1371/journal.pone.0332784.r001

**Authors:** Efrem Kentiba

29 Dec 2025

PONE-D-25-48289

Organized community sport participation in children and youth with physical disability: a scoping review protocol

PLOS One

Dear Dr. Pollock,

Thank you for submitting your manuscript to PLOS ONE. After careful consideration, we feel that it has merit but does not fully meet PLOS ONE’s publication criteria as it currently stands. Therefore, we invite you to submit a revised version of the manuscript that addresses the points raised during the review process.

Please submit your revised manuscript by Feb 12 2026 11:59PM. If you will need more time than this to complete your revisions, please reply to this message or contact the journal office at plosone@plos.org. When you’re ready to submit your revision, log on to https://www.editorialmanager.com/pone/ and select the ‘Submissions Needing Revision’ folder to locate your manuscript file.

Please include the following items when submitting your revised manuscript:

- A letter that responds to each point raised by the academic editor and reviewer(s). You should upload this letter as a separate file labeled ‘Response to Reviewers’.
- A marked-up copy of your manuscript that highlights changes made to the original version. You should upload this as a separate file labeled ‘Revised Manuscript with Track Changes’.
- An unmarked version of your revised paper without tracked changes. You should upload this as a separate file labeled ‘Manuscript’.

If you would like to make changes to your financial disclosure, please include your updated statement in your cover letter. Guidelines for resubmitting your figure files are available below the reviewer comments at the end of this letter.

If applicable, we recommend that you deposit your laboratory protocols in protocols.io to enhance the reproducibility of your results. Protocols.io assigns your protocol its own identifier (DOI) so that it can be cited independently in the future. For instructions see: https://journals.plos.org/plosone/s/submission-guidelines#loc-laboratory-protocols. Additionally, PLOS ONE offers an option for publishing peer-reviewed Lab Protocol articles, which describe protocols hosted on protocols.io. Read more information on sharing protocols at https://plos.org/protocols?utm_medium=editorial-email&utm_source=authorletters&utm_campaign=protocols.

We look forward to receiving your revised manuscript.

Kind regards,

Efrem Kentiba, PhD

Academic Editor

PLOS One

Journal Requirements:

When submitting your revision, we need you to address these additional requirements.

1. Please ensure that your manuscript meets PLOS ONE’s style requirements, including those for file naming. The PLOS ONE style templates can be found at

https://journals.plos.org/plosone/s/file?id=wjVg/PLOSOne_formatting_sample_main_body.pdf and

https://journals.plos.org/plosone/s/file?id=ba62/PLOSOne_formatting_sample_title_authors_affiliations.pdf.

2. Please include captions for your Supporting Information files at the end of your manuscript, and update any in-text citations to match accordingly. Please see our Supporting Information guidelines for more information: http://journals.plos.org/plosone/s/supporting-information.

3. If the reviewer comments include a recommendation to cite specific previously published works, please review and evaluate these publications to determine whether they are relevant and should be cited. There is no requirement to cite these works unless the editor has indicated otherwise.

[Note: HTML markup is below. Please do not edit.]

Reviewers’ comments:

Reviewer’s Responses to Questions

**Comments to the Author**

1. Does the manuscript provide a valid rationale for the proposed study, with clearly identified and justified research questions?

The research question outlined is expected to address a valid academic problem or topic and contribute to the base of knowledge in the field.

Reviewer #1: Yes

**********

2. Is the protocol technically sound and planned in a manner that will lead to a meaningful outcome and allow testing the stated hypotheses?

The manuscript should describe the methods in sufficient detail to prevent undisclosed flexibility in the experimental procedure or analysis pipeline, including sufficient outcome-neutral conditions (e.g. necessary controls, absence of floor or ceiling effects) to test the proposed hypotheses and a statistical power analysis where applicable. As there may be aspects of the methodology and analysis which can only be refined once the work is undertaken, authors should outline potential assumptions and explicitly describe what aspects of the proposed analyses, if any, are exploratory.

Reviewer #1: Partly

**********

3. Is the methodology feasible and described in sufficient detail to allow the work to be replicable?

Descriptions of methods and materials in the protocol should be reported in sufficient detail for another researcher to reproduce all experiments and analyses. The protocol should describe the appropriate controls, sample size calculations, and replication needed to ensure that the data are robust and reproducible.

Reviewer #1: Yes

**********

4. Have the authors described where all data underlying the findings will be made available when the study is complete?

The PLOS Data policy requires authors to make all data underlying the findings described in their manuscript fully available without restriction, with rare exception, at the time of publication. The data should be provided as part of the manuscript or its supporting information, or deposited to a public repository. For example, in addition to summary statistics, the data points behind means, medians and variance measures should be available. If there are restrictions on publicly sharing data—e.g. participant privacy or use of data from a third party—those must be specified.

Reviewer #1: No

**********

5. Is the manuscript presented in an intelligible fashion and written in standard English?

PLOS ONE does not copyedit accepted manuscripts, so the language in submitted articles must be clear, correct, and unambiguous. Any typographical or grammatical errors should be corrected at revision, so please note any specific errors here.

Reviewer #1: Yes

**********

6. Review Comments to the Author

Please use the space provided to explain your answers to the questions above and, if applicable, provide comments about issues authors must address before this protocol can be accepted for publication. You may also include additional comments for the author, including concerns about research or publication ethics.

You may also provide optional suggestions and comments to authors that they might find helpful in planning their study.

(Please upload your review as an attachment if it exceeds 20,000 characters)

Reviewer #1: The protocol addresses an important and timely topic with a clear structure and adherence to recognized guidelines (JBI, PRISMA-ScR), which is commendable. However, several aspects require refinement to enhance clarity and rigor. Key terms such as “organized community sport” and “physical disability” need precise operational definitions to avoid ambiguity, and the rationale for the age cutoff (up to 25 years) should be justified. The objectives should explicitly integrate the theoretical frameworks (RE-AIM and QPPF) mentioned later in the text, and the global scope requires more detail on how language diversity, cultural differences, and low-resource settings will be handled. The methods section should provide clearer strategies for grey literature screening, translation, and inter-rater reliability checks. Additionally, the exclusion of unorganized sport may limit cultural relevance, and anticipated limitations such as heterogeneity and publication bias should be acknowledged. Minor issues include typographic inconsistencies, citation formatting, and duplicate references. Overall, the protocol is promising but would benefit from stronger conceptual clarity, methodological transparency, and attention to global applicability.

Specific Comments

Title

How do you operationalize “organized” and “community” in global contexts where informal sports dominate? Will para-athletics and adaptive sports be treated differently from mainstream sports?

Abstract

The abstract lacks explicit mention of frameworks (RE-AIM, QPPF) in objectives, these appear later but should be upfront. Also, no mention of anticipated limitations (e.g., heterogeneity, lack of intervention trials)

Why was age cutoff set at 25 years? Is this consistent with WHO youth definition or arbitrary?

How will you handle mixed-disability cohorts where physical and cognitive disabilities coexist?

Introduction

Heavy reliance on prevalence data without linking to sport participation gaps quantitatively.

Mentions barriers but does not classify them systematically (e.g., socio-economic vs infrastructural).

Line 62, “Positive social experiences enhance participation”, needs stronger theoretical underpinning (Self-Determination Theory?).

Line 79, How will you differentiate between “quality participation” and mere attendance?

Will cultural factors (e.g., gender norms in sport) be analyzed?

Review questions

Second question (“barriers and facilitators”) is broad, should specify if these will be mapped to frameworks or thematic synthesis.

Will you assess barriers/facilitators quantitatively (frequency) or qualitatively (themes)?

How will you address intersectionality (e.g., gender + disability)?

Eligibility criteria

“Physical disability” definition is vague, should align with ICD or ICF classification.

Exclusion of “unorganized sport” may omit culturally relevant practices in LMICs.

Why exclude school-based therapy interventions but include school sports?

How will you handle hybrid programs (therapy + sport)?

Methods

Grey literature search strategy is ambitious but lacks detail on screening rigor.

No mention of language translation process, critical for global scope.

Will you use dual independent screening for grey literature?

How will you assess methodological quality (JBI critical appraisal tools?) even though scoping reviews typically do not?

References

Inconsistent referencing (authors used ‘ …et al’ after mentioning three authors in reference [2], whereas after mentioning six authors in references, [3, 10, 19, 21, …]. Also eight authors were mentioned in reference [16], reference number [13] and [15] are the same, please check all references in the entire protocol and format according to the journal’s guideline.

Supplementary files

Search strategy lacks Boolean logic clarity for complex queries

**********

7. PLOS authors have the option to publish the peer review history of their article (what does this mean?). If published, this will include your full peer review and any attached files.

If you choose “no”, your identity will remain anonymous but your review may still be made public.

**Do you want your identity to be public for this peer review?**  For information about this choice, including consent withdrawal, please see our Privacy Policy.

Reviewer #1: **Yes:** Victoria Drazdova

**********

[NOTE: If reviewer comments were submitted as an attachment file, they will be attached to this email and accessible via the submission site. Please log into your account, locate the manuscript record, and check for the action link "View Attachments". If this link does not appear, there are no attachment files.]

To ensure your figures meet our technical requirements, please review our figure guidelines: https://journals.plos.org/plosone/s/figures

You may also use PLOS’s free figure tool, NAAS, to help you prepare publication quality figures: https://journals.plos.org/plosone/s/figures#loc-tools-for-figure-preparation.

NAAS will assess whether your figures meet our technical requirements by comparing each figure against our figure specifications.

# Author comment: Author response to Decision Letter 1

**DOI:** 10.1371/journal.pone.0332784.r002

26 Feb 2026

February 16, 2025

Dear Academic Editor and Reviewer,

We thank the reviewer for their thoughtful and constructive edits and points to consider as well as the opportunity to submit a revised version of our manuscript. Please see our responses below each point raised in the review of the study protocol submission PONE-D-25-48289 “Organized community sport participation in children and youth with physical disability: a scoping review protocol.” Line numbers align with the file labeled ‘Revised Manuscript with Tracked Changes’.

Reviewer #1:

The protocol addresses an important and timely topic with a clear structure and adherence to recognized guidelines (JBI, PRISMA-ScR), which is commendable. However, several aspects require refinement to enhance clarity and rigor. Key terms such as “organized community sport” and “physical disability” need precise operational definitions to avoid ambiguity, and the rationale for the age cutoff (up to 25 years) should be justified. The objectives should explicitly integrate the theoretical frameworks (RE-AIM and QPPF) mentioned later in the text, and the global scope requires more detail on how language diversity, cultural differences, and low-resource settings will be handled. The methods section should provide clearer strategies for grey literature screening, translation, and inter-rater reliability checks. Additionally, the exclusion of unorganized sport may limit cultural relevance, and anticipated limitations such as heterogeneity and publication bias should be acknowledged. Minor issues include typographic inconsistencies, citation formatting, and duplicate references. Overall, the protocol is promising but would benefit from stronger conceptual clarity, methodological transparency, and attention to global applicability.

Response: We do not believe this scoping review is limited by publication bias. We have conducted a rigorous grey literature search, and we will include all publication types that meet our eligibility criteria.

Specific Comments

Title

How do you operationalize “organized” and “community” in global contexts where informal sports dominate?

Response: In the Introduction we have further clarified, The European Sports Charter defines sport as “all forms of physical activity which, through casual or organized participation, are aimed at maintaining or improving physical fitness and mental well-being, forming social relationships or obtaining results in competition at all levels”[1]. Within this definition, our review will focus on organized sports involving structured programs with regular planned practice and defined objectives (e.g., typically including skill development, physical fitness, teamwork, recreation and/or competition). See lines 35-37.

To operationalize “community”, the following has been added to the context section in the inclusion and exclusion criteria in Methods, Lines 148-152: This review will consider studies that implement organized sport participation programs for children and youth with physical disability in the community. Community settings will not be limited to specific geographical areas. They may include but will not be limited to indoor or outdoor recreation centres, fields, sports clubs, gymnasiums, and aquatic centres. We have removed the comment in Line 150 that there will be a global scope to remove misinterpretation that we will be able to capture a true global scope of community sports participation. Our aim is to focus on formal sports participation for children and youth with physical disability.

Will para-athletics and adaptive sports be treated differently from mainstream sports?

Response: Added to the concept section in the inclusion and exclusion criteria in Methods, Lines 138-140: Studies that include para-athletics, adaptive sports, and integrated programs in mainstream sports will be evaluated correspondingly through the lens of the QPPF and RE-AIM Frameworks.

Abstract

The abstract lacks explicit mention of frameworks (RE-AIM, QPPF) in objectives, these appear later but should be upfront.

Response: Added to the abstract, Lines 8-11: The objective of this scoping review is to identify evidence of evaluation of organized community sport participation opportunities for children and youth with physical disabilities through the lens of the RE-AIM and Quality Parasport Participation Frameworks.

Also, no mention of anticipated limitations (e.g., heterogeneity, lack of intervention trials).

Response: Added to the abstract, Lines 19-23: Anticipated limitations are the exclusion of clinical intervention trials and the exclusion of unorganized sport. The latter may limit cultural relevance and global scope, as informal sports could dominate in this context and be underrepresented in the literature. Languages will also be restricted to English, French, Portuguese and Spanish. No publication date restrictions will be placed on the search. We expect heterogeneity and consider this a strength.

Why was age cutoff set at 25 years? Is this consistent with WHO youth definition or arbitrary?

Response: Added to the participants section in the inclusion and exclusion criteria in Methods, Lines 120-122: This age cutoff is consistent with the WHO and United Nations’ definitions of youth[23,24]. Studies with participants (adults) outside of this age range will be included if at least 50% of the population sample is less than 25 years of age.

How will you handle mixed-disability cohorts where physical and cognitive disabilities coexist?

Response: Added to the participants section in the inclusion and exclusion criteria in Methods, Lines 124-126: Studies including mixed-disability cohorts, where physical, sensory and/or cognitive disabilities coexist, will be included if at least 50% of the population sample have a primary physical disability or sensory impairment.

Introduction

Heavy reliance on prevalence data without linking to sport participation gaps quantitatively.

Response: Added link to this gap in the Introduction, Lines 61-69: Children and youth with disabilities experience significant gaps in sport participation, taking part in less physical activity, recreation and sport than their typically developing peers and less likely to meet international guidelines for physical activity[5, 12-16]. Further, children and adolescents with disability participate less, and with less diversity, frequency and intensity, in team or non-team sports when compared to their peers with typical development[15,17]. They are also more likely to play informal or casual games at home than more formal or organized sports in their community, even if sports are their preferred activities[15,17].

Mentions barriers but does not classify them systematically (e.g., socio-economic vs infrastructural).

Response: Removed specific barriers from the Introduction, Lines 48-50, as these will be included in the data extraction tool and classified systematically in the final manuscript.

Line 62, “Positive social experiences enhance participation”, needs stronger theoretical underpinning (Self-Determination Theory?).

Response: Added to the Introduction, Lines 41-47: Positive social experiences from participation in sport for youth with physical disability is defined more comprehensively by the Quality Parasport Participation Framework (QPPF)[6]. This framework integrates insights from several theoretical underpinnings to define optimal or quality participation incorporating unique social benefits and well documented facilitators and barriers that impact physical activity and sport participation in this population.

Line 79, How will you differentiate between “quality participation” and mere attendance?

Response: Many studies may report attendance and not address a metric that will inform quality participation, and this will be noted. Quality participation will be described and evaluated through the constructs of the QPPF. Added to the Introduction, Lines 72-78: The Canadian Disability Participation Project (CDPP) is a national organization that aims to improve quality participation, differentiating from merely recording attendance, in community-based sport, optimizing outcomes of participation among children, youth and adults experiencing disability[18]. They developed the Quality Parasport Participation Framework (QPPF) to identify and describe quality experience in sport inclusive of six building blocks: autonomy, belongingness, challenge, engagement, mastery, and meaning[6,19].

Will cultural factors (e.g., gender norms in sport) be analyzed?

Response: We will report participation levels by sex and/or gender in our data extraction tool. Added sex and/or gender as a descriptor under population heading in the data extraction instrument in S3 Table, Line 340.

Review questions

Second question (“barriers and facilitators”) is broad, should specify if these will be mapped to frameworks or thematic synthesis.

Response: Added to the second review question in the Introduction, Lines 113-114: What are the perceived barriers and facilitators for community sport program implementation when mapped to the QPPF and RE-AIM framework?

Will you assess barriers/facilitators quantitatively (frequency) or qualitatively (themes)?

Response: Added to the concept section in the inclusion and exclusion criteria in Methods, Lines 144-146: Barriers and facilitators to participation will be mapped to the RE-AIM framework and the QPPF. They will be assessed quantitatively in terms of frequency in relation to the RE-AIM framework and qualitatively in accordance with the themes described in the QPPF.

How will you address intersectionality (e.g., gender + disability)?

Response: Added to data extraction in Methods, Lines 206-207: Themes or barriers specific to sex and/or gender will be captured in the data extraction tool and described.

Eligibility criteria

“Physical disability” definition is vague, should align with ICD or ICF classification.

Response: Added to the Introduction, Lines 54-59: The World Health Organization (WHO) describes disability as an inherent part of the human experience; the outcome of the interaction between an experienced health condition and/or impairment and circumstances associated with the environment and personal factors[9]. Within the context of the International Classification of Functioning, Disability and Health (ICF), a physical disability is a health condition or physical impairment that may limit activity or restrict participation[10].

Exclusion of “unorganized sport” may omit culturally relevant practices in LMICs.

Response: Added to the context section in the inclusion and exclusion criteria in Methods, Lines 148-152: This review will consider studies that implement organized sport participation programs for children and youth with physical disability in the community. Community settings will not be limited to specific geographical areas. They may include but will not be limited to indoor or outdoor recreation centres, fields, sports clubs, gymnasiums, and aquatic centres.

Why exclude school-based therapy interventions but include school sports?

Response: We will exclude both school-based therapy interventions and school sports. This is added to the context section in the inclusion and exclusion criteria in Methods, Lines 152-154: We will exclude studies that take place at home, in a clinical setting or school-based therapy interventions. Organized sports participation in a school setting will also be excluded due to underlying differences in program structure.

How will you handle hybrid programs (therapy + sport)?

Response: Added to the context section in the inclusion and exclusion criteria in Methods, Lines 154-156: Hybrid therapy (clinical) and sport programs will be included if there are community sport-related goals or if the aim is to support community sport participation.

Methods

Grey literature search strategy is ambitious but lacks detail on screening rigor.

Response: Added to the study/source of evidence section in Methods, Lines 188-197: Following the search, all identified records, including grey literature, will be collated and uploaded into Covidence (Veritas Health Innovation, Melbourne, Australia) and duplicates removed. Following a pilot test, titles and abstracts will then be screened by two independent reviewers for assessment against the inclusion criteria for the review. Potentially relevant papers will be retrieved in full. The full text of selected citations will be assessed in detail against the inclusion criteria by two independent reviewers. Reasons for exclusion of full-text papers that do not meet the inclusion criteria will be recorded and reported in the scoping review. Any disagreements that arise between the reviewers at each stage of the selection process will be resolved through discussion or with a third reviewer. The results of the search will be reported in full in the final scoping review and presented in a PRISMA flow diagram (S1)[28].

No mention of language translation process, critical for global scope.

Response: Added to the study/source of evidence section in Methods, Lines 198-199: We will include studies in English, French, Portuguese and Spanish in line with author fluency. This will be addressed as a limitation in the final manuscript.

Will you use dual independent screening for grey literature?

Response: Added to the study/source of evidence section in Methods, Lines 188-191: Following the search, all identified records, including grey literature, will be collated and uploaded into Covidence (Veritas Health Innovation, Melbourne, Australia) and duplicates removed. Following a pilot test, titles and abstracts will then be screened by two independent reviewers for assessment against the inclusion criteria for the review.

How will you assess methodological quality (JBI critical appraisal tools?) even though scoping reviews typically do not?

Response: Added in the Methods, Lines 214-221, All eligible studies included in the review will be critically appraised by two independent reviewers for methodological quality in the review using standardized critical appraisal instruments from JBI for experimental, quasi-experimental, observational and qualitative studies. A pilot test will be conducted of five studies to ensure consistency. If a team member has authored any studies included in our review, they will abstain from critical appraisal. Any disagreements that arise between the reviewers will be resolved through discussion or with a third reviewer. The results of critical appraisal will be reported in a table with accompanying narrative.

References

Inconsistent referencing (authors used ‘ …et al’ after mentioning three authors in reference [2], whereas after mentioning six authors in references, [3, 10, 19, 21, …]. Also, eight authors were mentioned in reference [16], reference number [13] and [15] are the same, please check all references in the entire protocol and format according to the journal’s guideline.

Response: Referencing has been edited for consistency and in the format according to PLOS ONE’s guidelines.

Supplementary files

Search strategy lacks Boolean logic clarity for complex queries.

Response: Our search strategy was designed by a health sciences librarian and is reported in accordance with the PRISMA 2020 guidelines that require a search strategy be reported exactly as run in the database. We have added a diagram to help clarify the complex Boolean logic at play in the strategy for the layperson in S2 Fig Search strategy Lines 331-332.

Have the authors described where all data underlying the findings will be made available when the study is complete?

Reviewer #1: No

Response: Added in Results and Discussion, Lines 228-232: We will share a narrative synthesis of the results and tables and/or graphs as appropriate in accordance with the review objectives and questions. All data underlying the scoping review (including search strategies, reference files at each stage and full data extraction and critical appraisal) will be deposited in the Open Science Framework: https://osf.io/dsny4.

Thank-you for considering our scoping review protocol revisions. We look forward to hearing from you.

Sincerely,

Karen Davies

krdavies@cw.bc.ca

Attachment Submitted filename: Response to Reviewers.docx

# Editor report: Decision Letter 1

**DOI:** 10.1371/journal.pone.0332784.r003

**Authors:** Efrem Kentiba

30 Apr 2026

Organized community sport participation for children and youth with physical disability: a scoping review protocol

PONE-D-25-48289R1

Dear Dr.  Pollock,

We’re pleased to inform you that your manuscript has been judged scientifically suitable for publication and will be formally accepted for publication once it meets all outstanding technical requirements.

Within one week, you’ll receive an e-mail detailing the required amendments. When these have been addressed, you’ll receive a formal acceptance letter and your manuscript will be scheduled for publication.

An invoice will be generated when your article is formally accepted. Please note, if your institution has a publishing partnership with PLOS and your article meets the relevant criteria, all or part of your publication costs will be covered. Please make sure your user information is up-to-date by logging into Editorial Manager at Editorial Manager® and clicking the ‘Update My Information’ link at the top of the page. For questions related to billing, please contact billing support.

If your institution or institutions have a press office, please notify them about your upcoming paper to help maximize its impact. If they’ll be preparing press materials, please inform our press team as soon as possible -- no later than 48 hours after receiving the formal acceptance. Your manuscript will remain under strict press embargo until 2 pm Eastern Time on the date of publication. For more information, please contact onepress@plos.org.

Kind regards,

Efrem Kentiba, PhD

Academic Editor

PLOS One

Additional Editor Comments (optional):

Reviewers’ comments:

# Editor report: Acceptance letter

**DOI:** 10.1371/journal.pone.0332784.r004

**Authors:** Efrem Kentiba

PONE-D-25-48289R1

PLOS One

Dear Dr. Pollock,

I’m pleased to inform you that your manuscript has been deemed suitable for publication in PLOS One. Congratulations! Your manuscript is now being handed over to our production team.

At this stage, our production department will prepare your paper for publication. This includes ensuring the following:

* All references, tables, and figures are properly cited

* All relevant supporting information is included in the manuscript submission,

* There are no issues that prevent the paper from being properly typeset

You will receive further instructions from the production team, including instructions on how to review your proof when it is ready. Please keep in mind that we are working through a large volume of accepted articles, so please give us a few days to review your paper and let you know the next and final steps.

Lastly, if your institution or institutions have a press office, please let them know about your upcoming paper now to help maximize its impact. If they’ll be preparing press materials, please inform our press team within the next 48 hours. Your manuscript will remain under strict press embargo until 2 pm Eastern Time on the date of publication. For more information, please contact onepress@plos.org.

You will receive an invoice from PLOS for your publication fee after your manuscript has reached the completed accept phase. If you receive an email requesting payment before acceptance or for any other service, this may be a phishing scheme. Learn how to identify phishing emails and protect your accounts at https://explore.plos.org/phishing.

If we can help with anything else, please email us at customercare@plos.org.

Thank you for submitting your work to PLOS ONE and supporting open access.

Kind regards,

PLOS ONE Editorial Office Staff

on behalf of

Dr. Efrem Kentiba

Academic Editor

PLOS One
